# High mortality in subjects with both diabetes and HIV in sub-Saharan Africa

**DOI:** 10.1097/QAD.0000000000001929

**Published:** 2018-09-03

**Authors:** Philip I. Burgess, Simon P. Harding, Petros C. Kayange, Joep van Oosterhout, Marta García-Fiñana, Gerald Msukwa, Theresa J. Allain

**Affiliations:** a Department of Eye and Vision Science, University of Liverpool, Liverpool, UK; b Malawi Liverpool Wellcome Trust Clinical Research Programme, Blantyre, Malawi; c College of Medicine, University of Malawi; d Dignitas International, Zomba Central Hospital, Zomba, Malawi; e Department of Biostatistics, University of Liverpool, Liverpool, UK; f Department of Medicine, Lions Sight First Eye Unit, Queen Elizabeth Central Hospital, Blantyre, Malawi; g Bristol Royal Infirmary, Bristol, UK.

Smit *et al.*[1] report that the mean age of people living with HIV in sub-Saharan Africa is increasing; the proportion suffering from at least one noncommunicable disease is predicted to rise. The authors are correct to highlight this important demographic change. There is a scarcity of data on the interaction between HIV and noncommunicable diseases. We performed the first prospective cohort study of diabetic retinopathy in sub-Saharan Africa [2,3]. In patients with both diabetes and HIV infection, we found a very high mortality rate but no increase in the rate of diabetic microvascular complications. Participants were systematically sampled from two hospital-based, primary care diabetes clinics. Glycaemic control, SBP, HIV status, urine albumin–creatinine ratio, and haemoglobin and serum lipid levels were assessed at baseline, 12 and 24 months. Retinopathy was graded at an accredited reading centre. Mortality was confirmed systematically either by death certificate or by key informants.

Of 357 people with diabetes included in the study, 50 were HIV positive (48 at baseline and two new diagnoses during the study). At baseline, HIV-positive participants demonstrated lower mean age (48.2 vs. 53.3 years; *P* = 0.015) and shorter duration of diabetes (2.8 vs. 4.4 years; *P* = 0.002) than HIV-negative individuals; a higher proportion of HIV-positive participants demonstrated raised urine albumin–creatinine ratio (50.0 vs. 31.6%; *P* = 0.015). Of 41 HIV-positive participants who underwent annual CD4^+^ cell count testing, 32 (78%), 25 (61%) and 14 (34%) individuals had a CD4^+^ cell count below 500, 350 and 200 cells/μl at any visit, respectively. At 24 months, 38 participants were assessed and nine had died (94% follow-up). Cumulative incidence of death amongst HIV-positive participants with diabetes at 12 and 24 months was 10% (1.7–18.3) and 18.1% (7.4–28.8), respectively (*n* = 50), compared with 5.4% at 24 months [2.8–8.0, 95% confidence interval (CI); *n* = 294] in HIV-negative participants with diabetes. In comparison, Malawi National HIV Programme data indicate a mortality rate of 5.1% 24 months after commencement of antiretroviral therapy [4]. In univariate analysis, death during the study was associated with proliferative diabetic retinopathy [odds ratio (OR) 6.47; 2.51–16.7; *P* = 0.0001], moderate visual impairment (OR 8.21; 2.48–27.1; *P* = 0.001) and HIV (OR 3.72; 1.54–9.00; *P* = 0.003).

At 24 months, two-step (or greater) progression of diabetic retinopathy was observed in 2/38 HIV-positive participants (5.3%) compared with 55/251 (21.9%) HIV-negative participants (*P* < 0.015 Fisher's exact). In multivariate logistic analysis, two-step progression of diabetic retinopathy was associated with HbA1c (OR 1.27, 95% CI 1.12–1.45), baseline grade of retinopathy (OR 1.39, 95% CI 1.02–1.91) and HIV infection (OR 0.16, 95% CI 0.03–0.78). The negative association between retinopathy progression and HIV infection may have been strongly influenced by the high mortality rate in people with HIV (people whose retinopathy would have otherwise progressed). No trend towards worsening renal function was identified at 24 months. Our data suggest that mortality in patients with both diabetes and HIV infection in Southern Malawi is very high. Previous studies have indicated that both HIV infection and antiretroviral therapies are associated with a vasculopathy which manifests as increased cardiovascular and cerebrovascular risk [5,6]. We did not find an increased rate of diabetic microvascular complications in persons with HIV in our cohort. Our results add to the growing literature on interaction between HIV and noncommunicable disease and highlight the urgent need for provision of integrated services for patients with diabetes and HIV infection.

## Acknowledgements

This work was funded by the Wellcome Trust via a Clinical PhD Fellowship (P.B. Grant number 094015/Z/10/A).

### Conflicts of interest

There are no conflicts of interest.
